# Iron Homeostasis-Related Parameters and Hepcidin/Ferritin Ratio: Emerging Sex-Specific Predictive Markers for Metabolic Syndrome

**DOI:** 10.3390/metabo14090473

**Published:** 2024-08-28

**Authors:** Baraah T. Abu AlSel, Abdelrahman A. Mahmoud, Elham O. Hamed, Noor A. Hakim, Abdulmajeed A. A. Sindi, Najlaa M. M. Jawad, Amani M. T. Gusti, Manal S. Fawzy, Noha M. Abd El-Fadeal

**Affiliations:** 1 Department of Pathology, Faculty of Medicine, Northern Border University, Arar 91431, Saudi Arabia; braatuma.aboalseel@nbu.edu.sa; 2 Faculty of Medicine, Sohag University, Sohag 82524, Egypt; abdelrahman.othman@med.sohag.edu.eg; 3 Sharaf Hospital, Ministry of Health, Hail 55211, Saudi Arabia; elhamomar@yahoo.com; 4 Department of Clinical Pathology, Faculty of Medicine, Sohag University, Sohag 82524, Egypt; 5 Department of Clinical Nutrition, Faculty of Applied Medical Sciences, King Abdulaziz University, Jeddah 21589, Saudi Arabia; ohakim@kau.edu.sa (N.A.H.); nalsini@kau.edu.sa (N.M.M.J.); 6 Department of Basic Medical Sciences, Faculty of Applied Medical Sciences, Al-Baha University, Al-Baha 65779, Saudi Arabia; asindi@bu.edu.sa; 7 Department of Medical Laboratory, Biochemistry, King Fahad Armed Forces Hospital, Jeddah 21159, Saudi Arabia; a_gusti2010@hotmail.com; 8 Department of Biochemistry, Faculty of Medicine, Northern Border University, Arar 91431, Saudi Arabia; 9 Center for Health Research, Northern Border University, Arar 91431, Saudi Arabia; 10 Unit of Medical Research and Postgraduate Studies, Faculty of Medicine, Northern Border University, Arar 91431, Saudi Arabia; 11 Medical Biochemistry and Molecular Biology Department, Faculty of Medicine, Suez Canal University, Ismailia 41522, Egypt; noha_abdelfadeal@med.suez.edu.eg; 12 Department of Biochemistry, Ibn Sina National College for Medical Studies, Jeddah 22421, Saudi Arabia

**Keywords:** metabolic syndrome, ferritin, hepcidin, HOMA-IR, insulin-sensitive, insulin resistance

## Abstract

Metabolic syndrome (MetS) is a worldwide public health challenge. Accumulating evidence implicates elevated serum ferritin and disruptions in iron metabolism as potential elements linked to an increased risk of MetS. This study investigates the relationship between iron homeostasis—including hepcidin levels, serum iron concentration, unsaturated iron-binding capacity (UIBC), and the hepcidin/ferritin (H/F) ratio—and MetS. In this descriptive cross-sectional study, 209 participants aged 24–70 were categorized into two groups: 103 with MetS and 106 without MetS. All participants underwent medical assessment, including anthropometric measures, indices of glycemic control, lipid profiles, and iron-related parameters. Participants were further stratified by the Homeostasis Model Assessment—Insulin Resistance index into three subgroups: insulin-sensitive (IS) (<1.9), early insulin resistance (EIR) (>1.9 to <2.9), and significant insulin resistance (SIR) (>2.9). Notable increments in serum ferritin and hepcidin were observed in the SIR group relative to the IS and EIR groups, with a significant association between metabolic parameters. The UIBC and serum ferritin emerged as significant predictors of MetS, particularly in men, with an area under the curve (AUC) of 0.753 and 0.792, respectively (*p* ≤ 0.001). In contrast, hepcidin was notably correlated with MetS in women, with an AUC of 0.655 (*p* = 0.007). The H/F ratio showed superior predictive capability for MetS across both sexes (at cutoff level = 0.67). Among women, this ratio had an AUC of 0.639 (*p* = 0.015), and for men, it had an AUC of 0.792 (*p* < 0.001). Hypertension proved an independent risk factor for MetS, affirming its role in metabolic dysregulation. The findings highlight a significant interconnection between iron homeostasis parameters and MetS, with sex-specific variations underscoring the importance of personalized diagnostic criteria. The crucial role of the H/F ratio and the UIBC as emerging predictive markers for MetS indicates their potential utility in identifying at-risk individuals. Further longitudinal research is essential to establish causality and explore the interplay between these biomarkers and MetS.

## 1. Introduction

The epidemic rise of metabolic syndrome (MetS) has emerged as a significant public health challenge, characterized by a cluster of conditions, including central obesity, hypertriglyceridemia, reduced high-density lipoprotein cholesterol (HDL-c) levels, elevated blood glucose, and hypertension [1,2]. These interrelated metabolic factors contribute to an elevated risk of cardiovascular disease, type 2 diabetes mellitus (T2DM), and other health complications, which lead to a significant societal and economic burden worldwide [3]. A combination of genetic, environmental, and lifestyle factors primarily drives this multifaceted syndrome, necessitating comprehensive research and multipronged intervention strategies [4].

Recent studies underscore the significant role of genetic predisposition in the development of MetS. Various genetic markers have been associated with different components of the syndrome, such as those involved in lipid metabolism, insulin resistance (IR), and inflammation pathways [5,6,7]. Additionally, lifestyle factors such as nutrition and physical activity are pivotal in determining the onset and progression of MetS. Diets rich in saturated fats, sugars, and processed foods significantly contribute to obesity and insulin resistance. On the other hand, balanced diets rich in fruits, vegetables, whole grains, and lean proteins have been shown to mitigate the risk of MetS [8]. Regular physical activity is equally crucial, as it helps maintain a healthy weight, improves lipid profiles, enhances insulin sensitivity, and reduces blood pressure [9,10]. Behavioral interventions focusing on these areas have demonstrated significant success in managing and even reversing MetS symptoms [11].

However, the prevalence of MetS can also be influenced by other factors beyond diet and lifestyle. Socioeconomic status, environmental pollutants, cultural practices, psychosocial stress, and healthcare policies are crucial in demographic and regional variations in MetS risk. Recognizing these multifaceted contributors underscores the need for comprehensive approaches to understanding and managing MetS globally [12,13].

The intricate interplay between metabolic dysregulation and various biomarkers has been a subject of intense research [14,15,16]. Among these biomarkers, hepcidin, a key regulator of iron metabolism, has raised scientific interest. Studies have proposed that hepcidin interacts with the cellular mechanisms underlying MetS, given its role in inflammation and its effect on iron distribution [17]. Moreover, other iron-related parameters, including serum ferritin (a surrogate marker for iron stores), have shown potential associations with MetS components [18,19,20]. Disruptions in regulating iron homeostasis may contribute to insulin resistance and the pathogenesis of MetS [21].

While the existing body of research has investigated the interrelations of metabolic abnormalities and their collective impact on global health, gaps remain in our understanding of these associations in certain ethnic and regional groups. This is particularly relevant when considering variances in genetic predispositions, lifestyle factors, and socioeconomic conditions that influence disease manifestation [2,22,23]. The rising prevalence of MetS in the Middle East necessitates a closer examination of the underlying metabolic disturbances specific to this region [24]. Given the unique genetic, environmental, and lifestyle factors prevalent in the Middle Eastern population, this study aims to investigate the association between iron metabolism—particularly the regulatory hormone hepcidin and serum ferritin levels—and the various features of MetS within this demographic. Specifically, we hypothesize that variations in iron metabolism markers correlate with different degrees of insulin sensitivity among the local population, including insulin-sensitive (IS) individuals and those with significant insulin resistance (SIR).

This study utilizes a cross-sectional design to compare these groups, aiming to provide insights that could lead to more tailored and effective management strategies for MetS in the Middle East. As current research predominantly centers on non-Middle Eastern populations, the related findings may not be directly applicable due to demographic and regional differences.

## 2. Materials and Methods

### 2.1. Study Participants

This cross-sectional, case-control study included consecutively identified individuals diagnosed with MetS, and others without MetS, who met the eligibility criteria. They were recruited between September 2018 and April 2019 at the tertiary Central Hospital in Arar, Saudi Arabia. Before starting the study, informed written consent was obtained from all enrolled participants. The study sample comprised 103 patients diagnosed with MetS and 106 participants presenting with no, or fewer than three, MetS criteria defined by the revised National Cholesterol Education Program Adult Treatment Panel III (NCEP-ATP III) guidelines [25]. A person is considered to have MetS when more than three of the following are present: hypertension ≥ 130/85 mm Hg; waist circumference measurements exceeding 92 cm for men and 87 cm for women (as established by the Saudi Abnormal Glucose Metabolism and Diabetes Impact Study) [26]; high fasting blood sugar (FBS) ≥ 100 mg/dL, or patients known to have T2DM; high total cholesterol (TC) ≥ 5.2 mmol/L; high triglyceride (TG) ≥ 1.7  mmol/L (and/or use hypolipidemic drugs); high low-density lipoprotein-cholesterol (LDL-c) ≥ 2.6 mmol/L; low HDL-c ≤ 1.03  mmol/L (men) or ≤1.3  mmol/L (women) or patients known to have dyslipidemia; elevated blood pressure (BP) characterized by a systolic BP of 130 mmHg or higher, and/or a diastolic BP of 85 mmHg or greater; or patients with a recognized history of hypertension and/or actively taking antihypertensive drugs. Based on the Homeostasis Model Assessment—Insulin Resistance (HOMA-IR) index calculation (https://www.thebloodcode.com/homa-ir-calculator) (accessed on 29 January 2024), we classified the participants into three groups: IS (≤1.9) (*n* = 106), early insulin resistance (EIR) (>1.9) (*n* = 43), and SIR (>2.9) (*n* = 60) [27,28].

The exclusion criteria for the study were as follows: (1) individuals under the age of 18; (2) smokers; (3) pregnant or breastfeeding women or women during the menstruation phase; (4) patients with pre-existing chronic conditions such as cancer or cardiovascular, liver, kidney, inflammatory, or blood disorders (except those with newly diagnosed T2DM or hypertension identified within the study setting); (5) those who had donated blood in the last six months; (6) patients on medications for other indications, including corticosteroids, oral contraceptive pills or other hormonal treatments and therapies that are acknowledged to alter the studied parameters concentrations; (7) individuals with missing data; and (8) those who were not willing to participate in the study.

Designated nurses from the participating hospital were tasked with collecting questionnaire data such as age, sex, and medical history of diabetes, hypertension, and dyslipidemia. They also conducted anthropometric assessments, recording height in centimeters, weight in kilograms, and circumferences of waist and hips using a Digital Pearson Scale (ADAM Equipment Inc., Oxford, CT, USA) [29]. Additionally, to promote reliability, participants had their resting blood pressure measured on three separate occasions, and blood samples were taken following established procedures. This ensured that demographic and clinical data for each participant were gathered accurately and comprehensively.

This research was rigorously scrutinized for compliance with the STROBE (Strengthening the Reporting of Observational Studies in Epidemiology) guidelines and was executed in adherence to the ethical principles set forth by the Institutional and National Research Committee and to be consistent with the Declaration of Helsinki and its subsequent modifications, as well as equivalent ethical norms. Ethical clearance was obtained from the Local Bioethics Committee.

### 2.2. Specimen Collection and Laboratory Investigations

Standard venous punctures were made to collect 5 mL overnight-fasting blood samples in trisodium EDTA tubes to provide complete blood picture evaluation and serum separator vacutainer Tubes II (Becton Dickinson Plymouth). After blood clotting, the latter tubes were centrifuged immediately at 700× *g* for 20 min. The separated serum was aliquoted into Eppendorf (500 µL per aliquot) and stored at −20 °C for subsequent biochemical assays. Here, FBS, TC, HDL-c, and TG levels were assessed in the serum using the enzymatic method with a Hitachi cobas^®^ 6000 automated chemistry analyzer (Roche Diagnostics Co., Mannheim, Germany). The LDL-c values were calculated using Friedewald’s equation [30], as all participants had a serum TG of <4.6 mmol/L. Fasting insulin levels were measured via electrochemiluminescence immunoassay (Cobas, Roche Diagnostics, Indianapolis, IN, USA). Insulin resistance was assessed using the HOMA-IR formula, calculated by multiplying fasting insulin (µU/L) × fasting glucose (nmol/L)/22.5 [31].

The hemoglobin A1c (HbA1c) was obtained using the cobas^®^ 6000 automated chemistry analyzer instrument based on turbidimetric inhibition immunoassay. Serum iron (Fe), unsaturated iron-binding capacity (UIBC), and ferritin levels were assayed using a Modular Analytics *p* or E170 analyzer. Serum hepcidin levels were analyzed using ELISA (a commercial Human Hepcidin ELIZA kit from Bioassay Technology Laboratory, Shanghai, China). During the laboratory work, quality control protocols were strictly adhered to. These included executing the necessary calibrators and controls before and during each assay to confirm the accuracy and reliability of the test results.

### 2.3. Statistical Analysis

Patient data were anonymized and encoded prior to analysis to ensure privacy. Continuous variables were presented as means ± standard error (SE) or means ± standard deviation (SD), as appropriate. These variables were compared using a Student’s *t*-test for two independent samples. When comparing more than two groups, one-way analysis of variance (ANOVA) was employed. For data that did not follow a normal distribution, the Kruskal–Wallis test was utilized. Post hoc analysis with the Bonferroni correction was performed for multiple comparisons. Categorical data were depicted as counts (percentages). These were analyzed using Chi-square or Fisher’s exact tests as appropriate. Logistic regression analysis was applied to compute odds ratios (OR) and 95% confidence intervals (CI) to assess the risk associated with the variables of interest. These were adjusted for notable confounding factors, including age, lipid profile, and glycemic control parameters. The capability of the measured parameters to discriminate between MetS and non-MetS was quantified through receiver operating characteristics (ROC) analysis, determining the area under the curve (AUC) and the optimal threshold values that provided the highest sensitivity and specificity. Additionally, 95% CIs for AUC values were calculated to ensure the robustness of the findings.

Power analysis with G*Power software (version 3.0.10) was used to validate the study sample. With the alpha error set at 0.05 and the specified sample size for each group, the study demonstrated a power of 94% to detect a minimum effect size of 0.5. Significance was established at *p*-values < 0.05. All data computations and statistical analyses were conducted using IBM SPSS Statistics for Windows (version 22.0, Armonk, NY, USA).

## 3. Results

### 3.1. Clinicolaboratory Characteristics of the Study Population

Table 1 summarizes the main clinical, anthropometrical, and biochemical features of the study participants. Our analysis encompassed two groups based on MetS status: 106 individuals without MetS and 103 patients meeting the MetS criteria. The mean age significantly differed between the groups, with a younger cohort in the non-MetS group (38.2 ± 11.1 years) compared with the MetS group (50.2 ± 9.1 years). This resulted in a significant *p*-value (<0.001). Within the non-MetS group, 56.6% were aged 40 years or below, contrasting with only 16.5% aged 40 or below in the MetS group. Participants older than 40 were predominantly observed in the MetS group (83.5%), compared with the non-MetS group (43.3%). Sex distribution was comparable between groups, with women constituting 66% of the non-MetS group and 68.9% of the MetS group (*p* = 0.661).

The prevalence of abdominal obesity was notably higher in the MetS group, with 91.3% testing positive, as opposed to 44.3% in the non-MetS group (*p* < 0.001). Dyslipidemia was universally present in the patients with MetS. This sharply contrasted with the non-MetS group, which was only present in 36.8% of participants (*p* < 0.001). Additionally, the biochemical profile showed significant disparities between the two groups, as was expected.

Iron metabolism parameters revealed no significant difference in Fe levels between the MetS and non-MetS groups (*p* = 0.07). The UIBC was slightly higher in the MetS group (51.5 ± 1.1 μmol/L) than in the non-MetS group (48.0 ± 1.36 μmol/L), approaching statistical significance (*p* = 0.05). Ferritin levels, a marker of iron storage, were higher in individuals with MetS (69.7 ± 7.09 ng/mL) than those without (44.5 ± 5.4 ng/mL), although this did not quite reach conventional levels of statistical significance (*p* = 0.05). Furthermore, hepcidin levels, critical in iron regulation, were significantly higher in the MetS group (24.2 ± 1.93 μIU/mL) than in the non-MetS group (17.2 ± 2.25 μIU/mL, *p* = 0.03). Overall, the data corroborate a clear association between MetS and clinical and biochemical alterations. In the context of MetS, elevated hepcidin levels, albeit without an appreciable difference in Fe levels, may suggest potential disruptions in iron homeostasis associated with metabolic dysregulation.

### 3.2. Association between HOMA-IR Index and Iron-Related Parameters

Table 2 illustrates the association between the HOMA-IR index and various iron-related parameters among the study participants. The participants were divided into three categories based on their HOMA-IR scores. A pronounced difference in mean Fe concentration was noted between the groups, with values decreasing as insulin resistance increased (*p* = 0.002). The IS group exhibited a mean Fe of 13.8 ± 0.48 µmol/L. This was higher than the EIR group’s mean of 11.8 ± 0.5 µmol/L and the SIR group’s mean of 9.2 ± 0.4 µmol/L. The unsaturated iron-binding capacity significantly varied among the different levels of insulin resistance (*p* = 0.001). The SIR group had a notably elevated UIBC (58.05 ± 1.8 µmol/L) compared with the IS (48.0 ± 1.36 µmol/L) and EIR groups (48.9 ± 1.3 µmol/L), suggesting that elevated insulin resistance may be associated with changes in iron transport capacity.

Ferritin levels significantly escalated when accompanied by increased levels of insulin resistance (*p* = 0.001). The gradient from the IS group, with a mean ferritin of 44.5 ± 5.4 ng/mL, to the EIR group at 57.8 ± 6.1 ng/mL, and culminating with the SIR group at 70.8 ± 5.9 ng/mL, suggests a positive correlation between iron stores and the degree of insulin resistance. Similarly, hepcidin levels also showed a progressive increase as insulin resistance decreased (*p* = 0.001); the IS group presented a mean hepcidin level of 17.2 ± 2.25 µIU/mL, which was lower than that in the EIR group (20.8 ± 1.2 µIU/mL), and substantially less than in the SIR group (25.6 ± 1.4 µIU/mL). These data support hepcidin’s potential role in modulating iron homeostasis.

### 3.3. Diagnostic Relevance of Circulatory Iron-Related Parameters for Metabolic Syndrome

**Figure 1:**
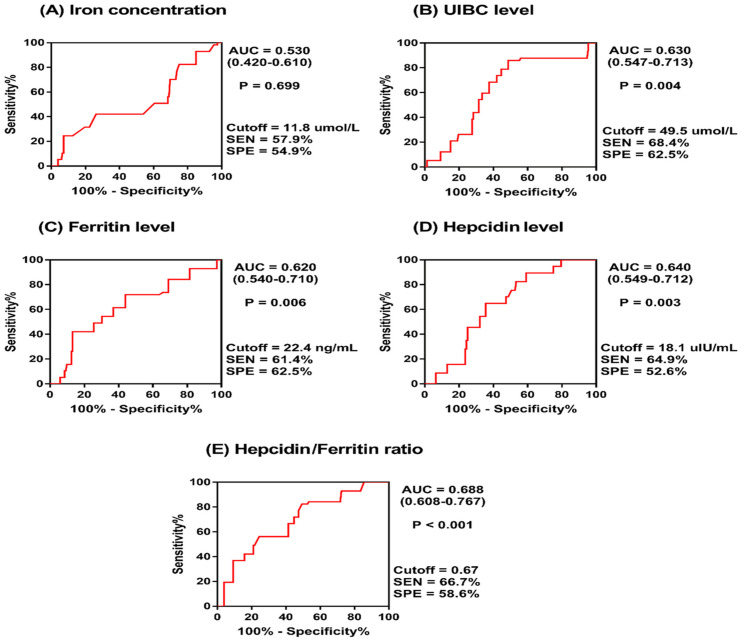
Receiver operating characteristics (ROC) curve for iron-related parameters predicting metabolic syndrome.

The diagnostic value of circulatory iron-related parameters for MetS was assessed using ROC curve analysis to calculate the AUC for each parameter. These analyses were conducted for the overall population (Figure 1) and separately for women and men (Table 3).

For the overall population cohort, the UIBC exhibited significant diagnostic value, with an AUC of 0.630 (*p* = 0.004), indicating a moderate ability to distinguish MetS cases. Ferritin also showed moderate diagnostic value, with an AUC of 0.620 (*p* = 0.006), while hepcidin levels were similarly predictive, with an AUC of 0.640 (*p* = 0.003). The hepcidin/ferritin (H/F) ratio demonstrated the most diagnostic utility, with an AUC of 0.688 (*p* < 0.001). However, Fe levels did not show significant diagnostic capacity (AUC = 0.530, *p* = 0.699).

On stratifying the study cohort by sex, there were notable differences in the diagnostic value of these parameters. Among women, hepcidin had a significant AUC of 0.655 (*p* = 0.007), followed by the H/F ratio with an AUC of 0.639 (*p* = 0.015). The UIBC, ferritin, and Fe levels did not demonstrate significant diagnostic potential in women, with *p*-values of 0.164, 0.355, and 0.581, respectively.

For men, the UIBC was discernable, with an AUC of 0.753 (*p* = 0.001), indicating a strong potential for MetS diagnosis. The H/F ratio also showed a high diagnostic capability, with an AUC of 0.792 (*p* < 0.001). In contrast, the Fe had a lower AUC of 0.349, which was significant (*p* = 0.043), pointing to a potential inverse relationship with MetS risk in men. Ferritin had an AUC of 0.248 (*p* = 0.001), suggesting an inverse correlation with MetS, and hepcidin showed no significant value in men (AUC = 0.535, *p* = 0.336).

These findings indicate that the UIBC and H/F ratio could have diagnostic relevance for MetS, with the H/F ratio being the most predictive and consistent across both sexes. Hepcidin showed better diagnostic performance in women, while the UIBC was more indicative for men. The varied AUC values between sexes highlight the importance of considering sex differences when evaluating the utility of iron-related biomarkers in MetS diagnosis.

### 3.4. Logistic Regression Analysis of Factors Influencing the Risk of Developing Metabolic Syndrome in the Study Population

Logistic regression analysis was used to evaluate the association of various clinicolaboratory factors with the risk of developing MetS (Table 4). Among all the variables analyzed, hypertension emerged as a robust predictor of MetS, with an OR of 3.957. This indicates that individuals with hypertension are approximately four times more likely to have MetS than those without hypertension (*p* < 0.001). The UIBC showed a significant B-value of 0.044 and is associated with a modestly higher risk of MetS (OR = 1.045, 95% CI: 1.004–1.088, *p* = 0.032). This association implies that for each unit increase in the UIBC, there is a 4.5% increase in the risk of an individual developing MetS, supporting a potential link between the UIBC levels and MetS risk. At the same time, the H/F ratio showed a significant OR of 1.068 (95% CI: 1.028–1.229, *p* = 0.043), suggesting a positive correlation with the risk of MetS. This significant finding hints at the potential role for the balance between iron regulatory mechanisms and iron stores in the development of MetS.

The participants’ FBS was also significantly associated with MetS, with an OR of 1.009 (95% CI: 1.002–1.015, *p* = 0.007). This suggests that each unit increase in FBS level is associated with a 0.9% increase in the risk of developing MetS. On the other hand, obesity and Fe levels did not show a significant association with MetS in this analysis, with *p*-values of 0.695 and 0.196, respectively. This lack of significance indicates that, within this study cohort, these parameters alone may not be reliable indicators of MetS risk or that other confounding factors may have influenced the parameters.

The constant (or intercept) of the logistic regression model showed a significant negative value (−5.804), indicating the log odds of being in the MetS category when all other predictors are held at zero. This highly significant constant (*p* < 0.001) reinforces the robustness of the model in predicting MetS status.

## 4. Discussion

In light of the increasing rates of obesity, prediabetes, and diabetes, it is crucial to investigate the elements that interact with MetS and its aspects. This is a matter of significant public health interest [32].

This research assessed the association between iron homeostasis-related parameters regarding the regulatory hormone hepcidin, serum ferritin, H/F ratio, Fe, and the UIBC with MetS among adults living in the northern region of Saudi Arabia. This area is part of the Middle East and North African (MENA) region, which has been documented to have a high incidence of MetS, a fact underscored by the findings of Al-Rubean et al.’s investigation [29]. Their data showed a MetS prevalence of 39.9%, with a gender breakdown of 45.0% in men and 35.4% in women. This was found by using the NCEP-ATP III criteria [25], the criteria that were also utilized in the present study. The researchers opted for this set of criteria over those proposed by the International Diabetes Federation (IDF) [33], as the IDF criteria include central obesity as a requisite. The use of IDF standards tends to report a lower prevalence of MetS, potentially overlooking individuals who exhibit other components of the syndrome despite not having central obesity. Consistent with the literature, our findings illustrate that iron metabolism has a substantial relationship with metabolic dysfunction [34,35,36,37,38].

The differential age distribution among non-MetS and MetS groups highlights the age-related increase in MetS prevalence (after 50, in particular), with the highest prevalence between 50 and 70 years [39,40,41]. Srivastav et al. suggested that “insulin resistance starts to manifest in the fourth decade of life concurrently with the development of hypertension, dyslipidemia, and obesity” [42]. Patients with MetS exhibited these physical traits significantly more than non-MetS participants (Table 1). Similarly, Momeni et al. indicated that the average age of their studied participants with T2DM was 56.5 ± 9.7, ranging from 30 to 82 years. They noted a reduction in serum ferritin levels following the regulation of hyperglycemia [43].

While no significant sex difference was observed, anthropometric measures such as BMI and abdominal obesity were starkly distinguished between the two groups, with remarkable elevations seen in the MetS cohort. These findings align with previous reports that underscore obesity as a leading feature of MetS [44]; however, our logistic regression analysis did not identify obesity as an independent predictor of MetS. This indicates the complexity of the syndrome, as other factors may play a more dominant role [7].

Moreover, the striking significance of hypertension in predicting MetS, as shown in our logistic regression model, should be emphasized. With an OR of approximately fourfold, hypertension is a critical component of MetS and a significant risk factor for cardiovascular diseases. This concurs with existing research [44,45,46].

Our findings present potential evidence for the involvement of iron regulation in MetS. Higher serum ferritin levels, indicative of increased iron stores, were found in the MetS group compared with the non-MetS group. However, this factor did not predict MetS when considered independently. Increased iron storage and the significant dysregulation of iron metabolism were related to higher insulin resistance, as demonstrated by the UIBC’s significant association with insulin resistance and MetS risk. This result aligns with prior research conducted in various other locations [47,48]. For example, Vaquero et al. reported that changes in iron transport and storage may occur in individuals who are overweight or obese, which coincides with the typical signs of insulin resistance [49]. In their work that correlated serum ferritin with MetS in eight Chinese cities, Wang et al. demonstrated a direct association between elevated serum ferritin levels and MetS and its components in multivariate analyses among men. These levels were independently associated with insulin resistance [50]. Similarly, Tran et al. reported the same observation in Vietnamese patients with MetS [41]. Previous studies showed that an altering iron metabolism in MetS could be due to increased transferrin saturation and/or increased hepcidin levels, reduced duodenal ferroportin expression, and impaired iron absorption [51].

Being a regulator of iron homeostasis, hepcidin might both influence and be influenced by inflammation, a hallmark of MetS [15]. This bidirectional relationship could explain the elevated hepcidin levels observed in this study’s MetS group. The lack of a significant association between Fe and MetS may appear counterintuitive, given the established connection between iron overload and metabolic disorders. However, it aligns with data suggesting that it is not the circulating iron per se but rather the body’s iron storage and regulatory capacity that play a role in the pathogenesis of MetS [34]. Studies showing controversial results may be due to the presence of genetic variation in genes responsible for the expression of proteins in the hepcidin axis, environmental exposure to pollutants affecting protein sharing in the iron homeostasis process, or dietary factors that may affect the level of iron and hepcidin [52].

The H/F ratio’s diagnostic power further supports this hypothesis, as it captures the dynamic between iron availability and storage better than ferritin or hepcidin alone [53]. Utilizing ratios as a metric is not a novel concept [54], yet its application as a predictive tool for MetS in at-risk populations would be innovative. While several ratios associated with iron status have been examined in distinct populations, such as blood donors, pregnant women, and individuals with cirrhosis, hemoglobinopathies, and neurodegenerative diseases [55], these have not been explored explicitly within the context of MetS. Given that our analysis identified an H/F ratio cutoff level of 0.67 for the entire cohort, we can hypothesize specific thresholds for males and females to identify a high risk of MetS while assuming other factors, such as diet and lifestyle, are controlled. Adapting this ratio-based method to individuals with MetS could offer significant advantages, including enhanced treatment guidance and a cost-effective, non-invasive, and proactive approach to healthcare.

Results from our logistic regression analysis indicated that the UIBC is a MetS predictor, corroborating the hypothesis that iron metabolism dysregulation could be involved in its pathogenesis [34]. While traditional views have positioned ferritin as a marker for body iron stores, our study highlights the role of the UIBC, suggesting that it could reflect subtle changes in iron status relative to metabolic alterations not detected by other measures.

Stratified analyses in our study have elucidated that sex plays a pivotal role in the relevance of circulatory iron-related biomarkers for diagnosing MetS. Further research is suggested to be conducted in different regions within Saudi Arabia to investigate the role of sex in these biomarkers. Interestingly, the UIBC held a more robust predictive value for MetS in male participants, while hepcidin levels performed better in the female population. Additionally, the H/F ratio was the most predictive and consistent across both sexes, underscoring its potential utility as a non-invasive biomarker. These findings align with previous literature that supports the roles of sex hormones in iron metabolism, offering a potential explanatory pathway for observed sex-specific associations in MetS [56,57,58]. Recognizing these sex-based differences in the diagnostic value of iron parameters is crucial. It emphasizes the need for gender-specific frameworks and interventions in both the clinical assessment and treatment strategies for MetS, which could be particularly effective in populations with high rates of sex-specific variations in MetS components [59,60,61,62,63,64]. This could complement the personalized medicine approach, where MetS management is tailored according to individual biomarker profiles [65,66].

Although our study contributes to understanding systemic iron regulation in MetS, it is equally important to consider that cellular iron regulation also plays a crucial role in the pathophysiological landscape of MetS [52]. At the cellular level, iron homeostasis involves a delicate balance of iron import, export, storage, and utilization processes, tightly regulated by proteins such as ferroportin, ferritin, and transferrin receptors. In MetS, chronic inflammation and oxidative stress can disrupt this balance, increasing intracellular iron levels. This is partly mediated by hepcidin-induced downregulation of ferroportin, resulting in decreased iron export from cells. The ensuing iron retention can exacerbate oxidative stress through the Fenton reaction, generating reactive oxygen species (ROS) and causing cellular damage [17]. Also, mitochondrial dysfunction, commonly associated with MetS, further complicates cellular iron metabolism. Inefficient iron–sulfur cluster and heme synthesis due to impaired mitochondrial function can disrupt cellular respiration, contributing to metabolic derangements characteristic of MetS [67]. Moreover, the regulatory mechanisms involving iron regulatory proteins (IRPs) are susceptible to dysregulation in the pro-inflammatory and oxidative environment of MetS. Altered IRP activity can lead to imbalanced expression of essential iron metabolism proteins, enhancing the risk of iron-mediated cellular damage and metabolic dysfunction [68].

The intricacies of how iron metabolism and homeostasis interplay with insulin resistance and MetS are complex and could have mutual associations [38]. Several theories propose that the relationship between iron metabolism and homeostasis may be mediated by insulin resistance and the increased oxidative stress caused by an iron surplus within the body (indicated by increased serum ferritin levels) [69]. Iron, a versatile transition metal, can transform into a highly reactive species that facilitates free radical generation, leading to the oxidative damage of cellular structures and tissues [68]. When there is a sustained imbalance between reactive oxygen species biogenesis and the antioxidant defenses, oxidative stress ensues. This stress is implicated in irregular alterations to cellular signaling and gene regulation, potentially progressing to pathological conditions such as insulin resistance [70]. Moreover, a collection of research has proposed that elevated ferritin levels may not solely reflect heightened iron reserves but may also signal ongoing inflammatory responses [71,72]. Furthermore, the body’s intricate iron metabolism, which encompasses the meticulous process of conserving and recycling this essential mineral, falls under the regulation of hepcidin [73]. Both iron stores and the need for erythropoiesis finely tune the production of hepcidin [17]. Conversely, MetS might contribute to iron dysregulation [74]. Thus, although connections between iron homeostasis-related parameters and MetS are apparent, the directional causality is challenging to pinpoint. For a more conclusive stance, future longitudinal research must ascertain whether these dysregulated variables are precursors to MetS or merely manifestations of underlying metabolic imbalances.

To sum up, our study implicates iron regulation as an essential aspect of MetS, both a potential contributing factor and a candidate for novel biomarkers. Our findings align with and contribute to an expanding body of research that suggests a complex interplay between iron homeostasis and metabolic health. Given that MetS and its components are modifiable risk factors for cardiovascular diseases, understanding this interplay is crucial for developing targeted interventions. Health practitioners should consider these relationships in prevention and treatment strategies for MetS, recognizing the potential benefits of monitoring iron-related biomarkers in at-risk patients.

Identifying emerging predictive markers (e.g., the H/F ratio), including several medical evaluation parameters, stratifying participants into multiple insulin resistance subgroups, and sex-based demarcation helps us understand the progression of MetS concerning iron parameters. It also provides a holistic view of the participant’s health status and strengthens the validity of the present findings. However, it is worth noting that caution is warranted in extrapolating these results without considering potential confounders such as diet, lifestyle, physical activity, medication regimens, socioeconomic status, environmental factors, and genetic predispositions. Also, the potential influence of hormonal fluctuations throughout the menstrual cycle in females may have introduced some variability in iron-related parameters. Hence, future studies should standardize the timing of blood sample collection to specific phases of the menstrual cycle, such as the follicular phase, to minimize the impact of menstrual blood loss and hormonal fluctuations on iron levels. Additionally, assessing hormonal levels could provide further insights into the interplay between hormones, iron metabolism, and MetS risk. Further longitudinal and interventional studies that include these confounders and encompass a more accurate assessment of body composition will be required to unravel the complex interactions and translate them into clinical practice.

## 5. Conclusions

This study delineates a significant association between serum levels of ferritin and hepcidin with MetS among the Saudi population. Individuals within the SIR subgroup notably presented with higher serum ferritin and hepcidin levels, underscoring a potential link between altered iron metabolism and insulin resistance. Collectively, these findings implicate iron regulatory proteins as contributors to metabolic disturbances and support the monitoring of iron status as a possible way of managing MetS. Further investigations are warranted to elucidate the causal relationships between MetS and iron metabolism-related proteins and underlying mechanisms, which could pave the way for targeted interventions in populations at risk of MetS.

## Figures and Tables

**Table 1:** Clinicolaboratory characteristics of the study participants.

Variables		Non-Metabolic Syndrome	Metabolic Syndrome	*p*-Value
Number		(n = 106)	(n = 103)	
Age	Mean ± SD	38.2 ± 11.1	50.2 ± 9.1	<0.001
	≤40 years	60 (56.6)	17 (16.5)	<0.001
	>40 years	46 (43.3)	86 (83.5)	
Sex	Female	70 (66)	71 (68.9)	0.661
	Male	36 (34)	32 (31.1)	
Weight, kg	Mean ± SD	76.9 ± 16.7	95.3 ± 17.7	<0.001
Height, cm	Mean ± SD	160.5 ± 8.2	161.9 ± 10.6	0.287
BMI, Kg/m^2^	Mean ± SD	30.1 ± 7.06	36.3 ± 5.7	<0.001
Abdominal obesity	Negative	59 (55.7)	9 (8.7)	<0.001
	Positive	47 (44.3)	94 (91.3)	
Hypertension	Negative	95 (89.6)	33 (32.0)	<0.001
	Positive	11 (10.4)	70 (68.0)	
Diabetes mellitus	Negative	97 (91.5)	39 (37.9)	<0.001
	Positive	9 (8.5)	64 (62.1)	
Dyslipidemia	Negative	67 (63.2)	0 (0.0)	<0.001
	Positive	39 (36.8)	103 (100)	
TC, mmol/L mean ± SE		4.50 ± 0.08	5.35 ± 0.10	<0.001
TG, mmol/L mean ± SE		1.14 ± 0.04	2.30 ± 0.10	<0.001
LDL-c, mmol/L mean ± SE		2.62 ± 0.07	3.15 ± 0.08	<0.001
HDL-c, mmol/L mean ± SE		1.3 ± 0.03	1.02 ± 0.02	<0.001
HOMA-IR mean ± SE		1.26 ± 0.09	2.11 ± 0.1	<0.001
FBS, mg/dL mean ± SE		97.8 ± 1.70	174 ± 2.3	<0.001
HbA1c% mean ± SE		2.9 ± 0.05	7.0 ± 0.17	<0.001
Insulin, μLU/mL mean ± SE		3.90 ± 0.38	10.4 ±1.23	<0.001
Fe, μmol/L mean ± SE		13.8 ± 0.48	12.14 ± 0.39	0.07
UIBC, μmol/L mean ± SE		48.0 ± 1.36	51.5 ± 1.1	0.05
Ferritin, ng/mL mean ± SE		44.5 ± 5.4	69.7 ± 7.09	0.05
Hepcidin, μLU/mL mean ± SE		17.2 ± 2.25	24.2 ± 1.93	0.03

Data are presented as frequencies (percentages) or mean ± standard deviations/errors (SD/SE). Fisher’s exact or Chi-square tests were used for qualitative variables, and Student’s *t*-test was used for quantitative data. Abdominal obesity: waist circumference ≥ 92 cm in men and ≥87 cm in women. Significance was set at *p*-values < 0.05. BMI: body mass index, TC: total cholesterol, TG: triacylglycerol, LDL-c: low-density lipoprotein-cholesterol, HDL-c: high-density lipoprotein-cholesterol, HOMA-IR: homeostasis model assessment of insulin resistance, FBS: fasting blood sugar, HbA1c: hemoglobin A1c, Fe: serum iron concentration, UIBC: unsaturated iron-binding capacity.

**Table 2:** Association of HOMA-IR index and iron-related parameters.

Variables	IS (n = 106)	EIR (n = 43)	SIR (n = 60)	*p*-Value
Fe (μmol/L)	13.8 ± 0.48	11.8 ± 0.5	9.2 ± 0.4	0.002 ^a^
UIBC (μmol/L)	48.0 ± 1.36	48.9 ± 1.3	58.05 ± 1.8	0.001 ^ab^
Ferritin (ng/mL)	44.5 ± 5.4	57.8 ± 6.1	70.8 ± 5.9	0.001 ^a^ 0.01 ^b^
Hepcidin (μLU/mL)	17.2 ± 2.25	20.8 ± 1.2	25.6 ± 1.4	0.001 ^a^ 0.013 ^b^

Data are presented as mean ± standard error. One-way ANOVA and Kruskal–Wallis tests were applied, followed by the Bonferroni multiple comparison test. ^a^ SIR compared to IS, ^b^ SIR compared to EIR. Subjects were classified into three groups according to their HOMA-IR index: insulin-sensitive (IS) (≤1.9), early insulin resistance (EIR) (>1.9), and significant insulin resistance (SIR) (>2.9). Significance was set at *p*-values < 0.05. Fe: serum iron concentration, UIBC: unsaturated iron-binding capacity.

**Table 3:** Evaluation of circulatory iron-related parameters in diagnosing metabolic syndrome, stratified by sex.

Variable	Overall (n = 209)		Women (n = 141)		Men (n = 68)	
	AUC	*p*-Value	AUC	*p*-Value	AUC	*p*-Value
Fe, μmol/L	0.530	0.699	0.531	0.581	0.349	0.043
UIBC, μmol/L	0.630	0.004	0.579	0.164	0.753	0.001
Ferritin, ng/mL	0.620	0.006	0.447	0.355	0.248	0.001
Hepcidin, μLU/mL	0.640	0.003	0.655	0.007	0.535	0.336
H/F ratio	0.688	<0.001	0.639	0.015	0.792	<0.001

Significance was set at *p*-values < 0.05. Fe: serum iron concentration, UIBC: unsaturated iron-binding capacity, H/F ratio: hepcidin/ferritin ratio, AUC: area under the curve.

**Table 4:** Logistic regression analysis for clinical/laboratory parameters and risk of metabolic syndrome.

Variable	OR	95% CI	*p*-Value
Obesity	0.832	0.333–2.082	0.695
Hypertension	3.957	1.840–8.508	<0.001
Fe, μmol/L	1.066	0.968–1.174	0.196
UIBC, μmol/L	1.045	1.004–1.088	0.032
H/F ratio	1.068	1.028–1.229	0.043
FBS	1.009	1.002–1.015	0.007
Constant	0.003		<0.001

The dependent variable was metabolic syndrome [a binary variable (0, 1)], whereas independent variables were Fe, UIBC, hepcidin/ferritin ratio, FBS [continuous variables (0–1)], as well as obesity and hypertension [binary variables (0, 1)]. Significance was set at *p*-values < 0.05. OR = odds ratio, 95% CI = 95% confidence interval, Fe = serum iron concentration, UIBC: unsaturated iron-binding capacity, H/F: hepcidin/ferritin ratio, FBS: fasting blood sugar.

## Data Availability

The original contributions presented in the study are included in the article, further inquiries can be directed to the corresponding author.
